# Supplementary material for: MATRIX: rapid quantification of total and active microbial cells with single-cell phenotypes for environmental microbiomes
Source: mSystems. 2026 Jul 15;11(8):e00308-26. doi: 10.1128/msystems.00308-26 (PMC13483317; doi:10.1128/msystems.00308-26)
Supplement: Supplementary file 1 [file msystems.00308-26-s0001.pdf]

Supplemental Information

MATRIX: Rapid Quantification of Total and Active Microbial Cells with Single-Cell Phenotypes for Environmental Microbiomes

Milena Gonzalo[1], Xipeng Liu[1], Yann S. Dufour[2], and Ashley Shade[1]

[1] Université Lyon 1, CNRS, INRAE, LEM, UMR 5557, UMR 1418 Villeurbanne, France

[2]DatInsights, 69003, Lyon, France

Supplemental Figures 1-3

Supplemental Model Parameters

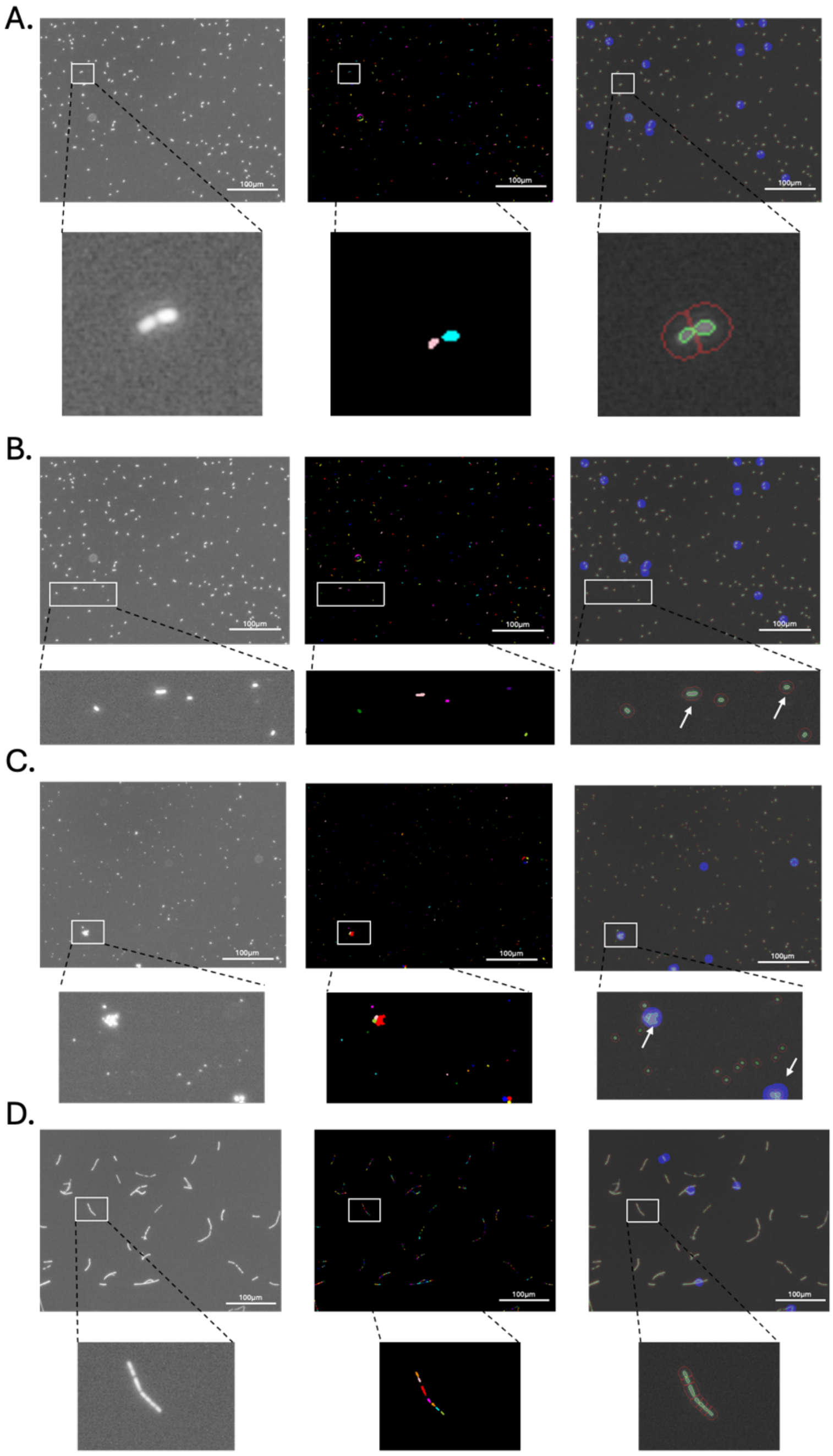

Figure S1. Examples of the accuracy of the detection and watershed segmentation protocol for different bacterial cells as applied by the process_quantom automated image analysis. For each panel, the full-size images are on top, and the zoomed and cropped subsection are at the bottom. The first column presented per panel is the raw fluorescence SYBR-stained image, the second column shows object detection, and the third column shows perimeter (green outline) and fluorescent signal bleeding (red outlines). A. Segmentation of two *Dyella koreensis* sp cells that are side-by-side. B. Segmentation of differently sized and shaped *Dyella koreensis* sp cells in a mixed-growth stage culture. C. Segmentation of an aggregation of *Mycobacterium mucogenicum* cells sp. D. Segmentation of chains of *Bacillus proteolyticus*.

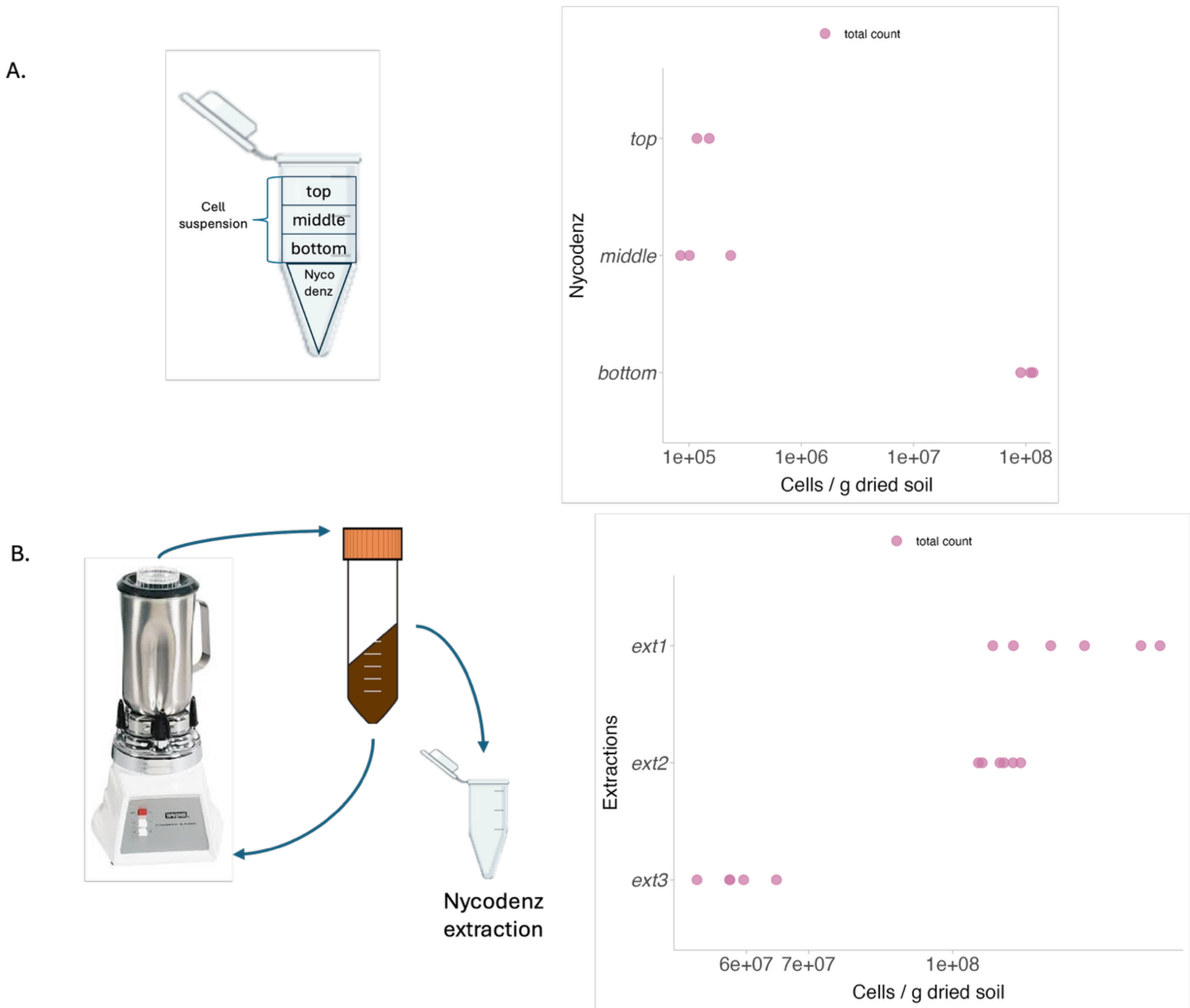

Figure S2. Evaluating the recovery of bacterial cells after separation from soil within the MATRIX pipeline. A. Most cells were recovered from the bottom layer of the cell suspension after density gradient separation with Nycodenz. Debris pelleted at the bottom of the tube. B. We performed a series of three consecutive soil blends to assess cell recovery and determined that the most cells were recovered after two blends and combined Nycodenz extractions. "Ext" is the consecutive extraction/blend step.

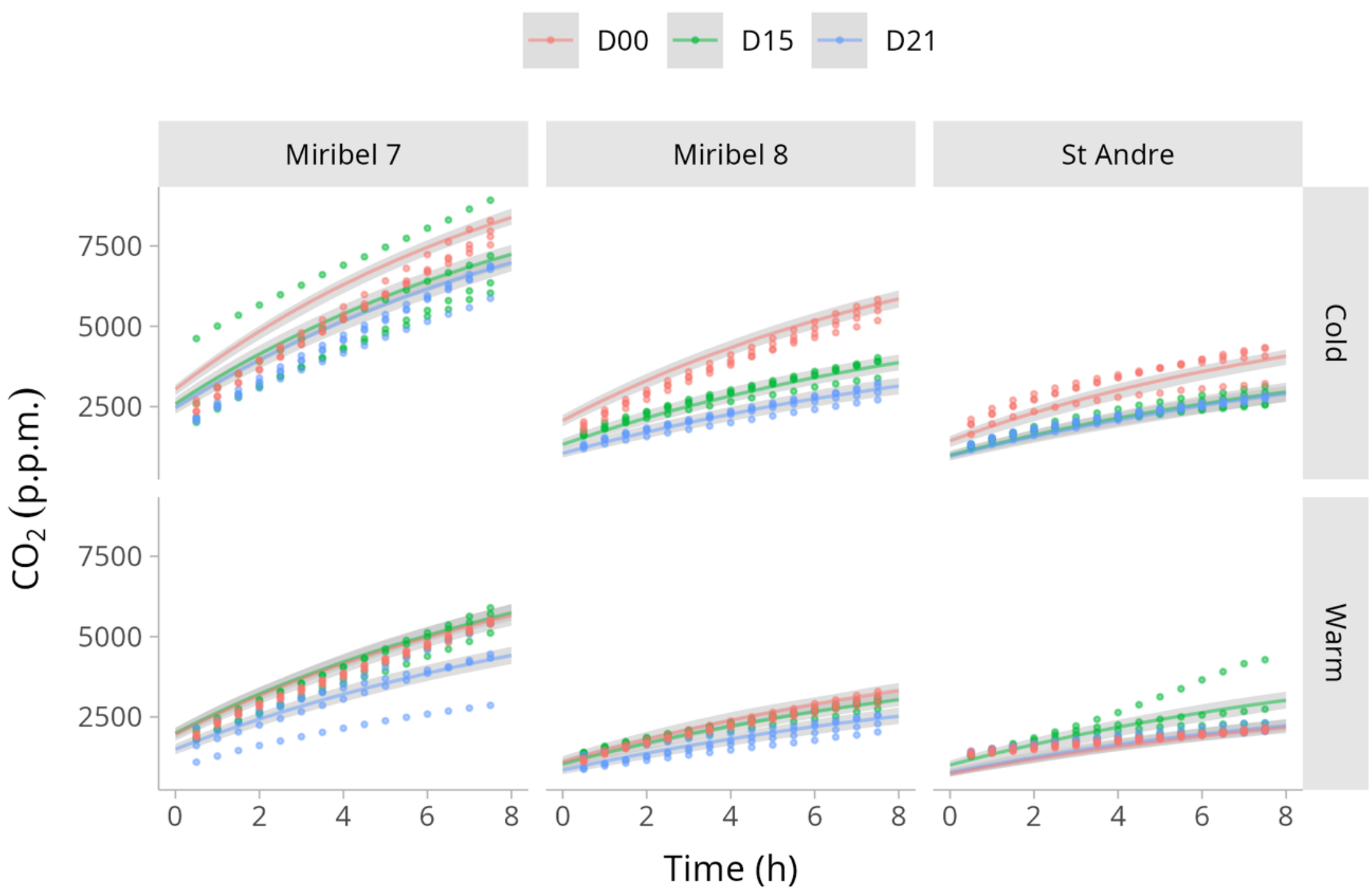

Figure S3. First-order kinetic fits of CO2 production (ppm) as a function of time (h) for different soil samples. Soil respiration was quantified in three soils during both the cold season (soil temperature, 4-7 °C) and the warm season (19-21 °C). D00, D15, and D21 (red, green, and blue, respectively) denote the duration of laboratory acclimation in days. Respiration rates were measured in triplicate for each sample. Points indicate observed measurements, whereas lines represent the median and 95% CrI of the posterior distributions for each combination of factors. The fitted rate constants were used to estimate metabolic activity from the temporal dynamics of CO2 accumulation during incubation. The model fitted to the data is described in the supplementary materials.

Supplementary materials: Rapid quantification of total and active environmental bacteria using automated image analysis: insights for single cells, populations, and communities

Milena Gonzalo, Xipeng Liu, Yann S. Dufour, and Ashley Shade

1 Figure 1B: Optical Density vs. Cell Concentration

We model the relationship between optical density (OD) and cell concentration using a non-linear mixed-effects model. Let n index the observations and j index the bacterial strains.

1.1 Model definition

The expected optical density for the n-th observation of strain j is given by:

$$OD_n = \beta_{0,j} + \beta_{1,j} \cdot (\mathrm{C}_n)^{\beta_{2,j}} + \epsilon_n \tag{1}$$

where:

- OD_n is the measured optical density for observation n.
- $\beta_{0,j}$ is the intercept parameter specific to strain j.
- $\beta_{1,j}$ is the slope coefficient specific to strain j.
- $\beta_{2,j}$ is the exponent coefficient specific to strain j.
- C_n is the measured cell concentration for observation n.
- ϵ_n is the residual error term drawn from a normal distribution, $\mathcal{N}(0, \sigma)$.

The parameters $\beta_{0,j}$, $\beta_{1,j}$, and $\beta_{2,j}$ vary by strain. We specify the following hierarchical priors for the strain-level deviations:

$$\beta_{0,j} \sim \mathcal{N}(\mu_{\beta_0}, \sigma_{\beta_0}) \tag{2}$$

where μ_{β_0} is the population-level mean intercept and σ_{β_0} is the standard deviation of the intercept across strains.

We treat the strain-specific values as draws from a normal distribution and a uniform distribution respectively:

$$\beta_{1,j} \sim \mathcal{N}(0, 0.1) \tag{3}$$

$$\beta_{2,j} \sim \mathrm{Uniform}(0, 1) \tag{4}$$

1.2 Prior Specifications

Specific informative priors Were applied to the population-level means and residual standard deviation with truncation boundaries as specified:

$$\mu_{\beta_0} \sim \mathcal{N}(0, 0.1), \quad \text{truncated to } [0, 0.2] \tag{5}$$

$$\sigma_{\beta_0} \sim \mathcal{N}(0, 0.1), \quad \text{truncated to } [0, 1] \tag{6}$$

$$\sigma \sim \mathcal{N}(0, 0.05), \quad \text{truncated to } [0, 0.1] \tag{7}$$

2 Figure 1C: Proportion of Active Cells

We model the proportion of active cells in the population using a linear mixed-effect model. Let n index the observations, j index the bacterial strains, and t the sampling time.

2.1 Model Definition

The cell counts in each sample are modeled using a negative binomial distribution parameterized by the log-mean and a dispersion parameter. The likelihood contribution for each observation is:

$$y_n^{\text{total}} \sim \text{NegativeBinomial}(\exp(\mu_n^{\text{total}}), \phi) \tag{8}$$

$$y_n^{\text{active}} \sim \text{NegativeBinomial}(\exp(\mu_n^{\text{active}}), \phi) \tag{9}$$

where NegativeBinomial denotes the second parameterization of the negative binomial distribution (mean-scale), such that $\text{Var}(y) = \mu + \frac{\mu^2}{\phi}$, and for each observation n:

- y_n^{total} is the measured total cell count.
- y_n^{active} is the active total cell count.
- μ_n^{total} is the log-mean for the total cell count.
- μ_n^{active} is the log-mean for the total cell count.
- ϕ is the common dispersion parameter.

The log-means are constructed from linear sums of fixed and random effects:

$$\mu_n^{\text{total}} = \mu_{j,t}^{\text{total}} + \epsilon_n \tag{10}$$

$$\mu_n^{\text{active}} = \mu_{j,t}^{\text{total}} + \log(p_{j,t}^{\text{active}}) + \epsilon_n \tag{11}$$

where:

- $\mu_{j,t}^{\text{total}}$ is the log of the mean of total cell count for each strain at each sampling time.
- $\log(p_{j,t}^{\text{active}})$ is the log of proportion of active cells for each strain at each sampling time.
- ϵ_n is the residual error term drawn from a normal distribution, $\mathcal{N}(0, \sigma)$.

We impose a constraint $\log(p_{j,t}^{\text{active}}) \leq 0$ to ensure that the proportion of active cells is bounded to [0, 1].

2.2 Prior Specifications

Specific informative priors Were applied to the population-level means and residual standard deviation with truncation boundaries as specified:

$$\mu_{j,t}^{\text{total}} \sim \text{Uniform}(-\infty, +\infty) \tag{12}$$

$$\log(p_{j,t}^{\text{active}}) \sim \text{Uniform}(-\infty, 0] \tag{13}$$

$$\epsilon_n \sim \mathcal{N}^+(0, \sigma) \tag{14}$$

$$\sigma \sim \text{Uniform}(0, \infty) \tag{15}$$

$$\phi \sim \text{Inverse-Gamma}(0.4, 0.3) \tag{16}$$

3 Figure 2D: Cell Volume vs. Growth Rate

We model growth rate as a smooth function of time using a generalized additive model (GAM), and cell volume as a function of growth rate using a linear mixed-effect model. Let n index the observations, j index the bacterial strains, and t the sampling time.

3.1 Model Definition

The cell concentrations in each sample are modeled using a log-normal distribution parameterized by the mean and standard deviation. The likelihood contribution for each observation is:

$$C_n \sim \text{LogNormal}(\mu_n^{\text{conc}}, \sigma^{\text{conc}}) \tag{17}$$

where for each observation n:

- C_n is the measured cell concentration.
- μ_n^{conc} is the mean concentration.
- σ^{conc} is the sampling error.

The estimated growth rate mean ($\mu_{j,t}$) and standard deviation ($\sigma_{j,t}$) for each strain at each time point is calculated from the first derivative of the posterior smooth estimates of the mean concentration to obtain the sampling distribution for the growth rate ($r_{j,t}$):

$$r_{j,t} \sim \mathcal{N}(\mu_{j,t}^r, \sigma_{j,t}^r) \tag{18}$$

We model cell volume as a linear function of the estimated growth rate while propagating the uncertainty. The likelihood contribution for each observation is:

$$V_n \sim \mathcal{N}(\mu_n^{\text{vol}}, \sigma^{\text{vol}}) \tag{19}$$

where for each observation n:

- V_n is the measured cell volume.
- μ^{vol} is the mean volume.
- σ^{vol} is the volume sampling error.

The concentration and volume means are constructed from linear sums of fixed effects:

$$\mu_n^{\text{conc}} = \mu_j^{\text{conc}} + s(t)_j \tag{20}$$

$$\mu_n^{\text{vol}} = \mu_j^{\text{vol}} + \beta_j r_{j,t} \tag{21}$$

where:

- μ_j^{conc} is the intercept of mean cell concentration for each strain.
- $s(t)_j$ is a smooth function of time regularized by a penalty on the wiggliness (integrated squared second derivative) for each strain.
- μ_j^{vol} is the intercept of mean cell volume for each strain.
- β_j is the slope of the linear relationship for each strain.

3.2 Prior Specifications

Specific informative priors Were applied to the population-level means and residual standard deviation with truncation boundaries as specified:

$$\mu_j^{\text{conc}} \sim \text{Uniform}(-\infty, +\infty) \tag{22}$$

$$\mu_j^{\text{vol}} \sim \text{Uniform}(-\infty, +\infty) \tag{23}$$

$$\beta_j \sim \text{Uniform}(-\infty, +\infty) \tag{24}$$

$$\sigma^{\text{conc}} \sim \text{Half-Student-t}(3, 0, 2.5) \tag{25}$$

$$\sigma^{\text{vol}} \sim \text{Half-Student-t}(3, 0, 2.5) \tag{26}$$

4 Figure 3B: Proportion of Active Cells

We model the proportion of active cells in the population using the same model as Figure 1C.

5 Figure 4B: Proportion of Active and Lysozyme Resistant Cells

We model the proportion of active and lysozyme resistant cells in the population using a non-linear mixed-effect model. Let n index the observations and j index the sampling sites.

5.1 Model Definition

The observed counts in each sample are modeled using a negative binomial distribution parameterized by the log-mean and dispersion parameter. The likelihood contribution of each observation is:

$$C_n \sim \text{NegativeBinomial}(\exp(\mu_n), \phi) \tag{27}$$

where for each observation n:

- C_n is the measured cell concentration.
- μ_n is the log-mean concentration.
- ϕ is the common dispersion parameter.

The total cell population can be divided into four categories: (i) active and lysozyme resistant (RA), (ii) inactive and lysozyme resistant (RI), (iii) active and lysozyme sensitive (SA), (iv) inactive and lysozyme sensitive (SI). Therefore, the total population log-mean for each sampling site can be written as the sum of the proportion for each category:

$$\mu_n = \mu_j^{\text{total}} + log(1 - p_j^{\text{RI}} - p_j^{\text{SA}} - p_j^{\text{SI}}) \tag{28}$$

$$p_j^{\text{RI}} + p_j^{\text{RA}} + p_j^{\text{SA}} + p_j^{\text{SI}} = 1 \tag{29}$$

where, the proportional contribution of each category is indexed to the experimental design.

5.2 Prior Specifications

Specific informative priors Were applied to the population-level means and dispersion as specified:

$$\mu_j^{\text{total}} \sim \text{Uniform}(-\infty, \infty) \tag{30}$$

$$p_j^{\text{cat}} \sim \text{Uniform}(0, 1) \tag{31}$$

$$\phi \sim \text{Half-Student-t}(3, 0, 2.5) \tag{32}$$

6 Figure 5: Respiration vs. Total RSG Fluorescence

We model the CO_2 respiration rates as a function of time, cell concentration, and moisture content using a non-linear mixed-effects model. We model the total RSG fluorescence using a linear mixed-effect model and fit a linear relation ship between the log transform of respiration parameters and the total fluorescence. Let n index the observations, j index the sampling sites, and d the sampling dates.

6.1 Model Definition

The likelihood for the CO_2 response for each observation n is:

$$\text{CO}_{2,n} \sim \mathcal{N}(\mu_n, \sigma^{\text{resp}}) \tag{33}$$

The mean μ_n is defined by the non-linear equation:

$$\mu_n = C_{j,d} \cdot (1 - M_{j,d}) \cdot (1 - \exp(-K_{j,d} \cdot (1 - M_{j,d}) \cdot (t + D_{j,d}))) \tag{34}$$

where:

- t is the time variable.
- $M_{j,d}$ is the measured moisture content.
- $C_{j,d}$ is the asymptotic concentration parameter specific to j, d.
- $K_{j,d}$ is the rate constant parameter specific to j, d.
- $D_{j,d}$ is the time delay parameter specific to j, d.
- σ is the residual standard deviation.

The parameters $C_{j,d}$, $K_{j,d}$, and $D_{j,d}$ are estimated without an intercept (fixed effects by group).

The likelihood for the total RSG fluorescence for each observation n is:

$$F_{j,d} \sim \text{LogNormal}(\mu^{\text{fluo}}_{j,d}, \sigma^{\text{fluo}}) \tag{35}$$

where:

- $\mu^{\text{fluo}}_{j,d}$ is log-mean of the total fluorescence.
- σ^{fluo} is the fluorescence sampling error.

The likelihood of the logs of the respiration parameters as a function of total fluorescence are:

$$\log(K_{j,d}) \sim \mathcal{N}(\mu^{\text{K}} + \beta^{K} F_{j,d}, \sigma^{\text{K}}) \tag{36}$$

$$\log(C_{j,d}) \sim \mathcal{N}(\mu^{\text{C}} + \beta^{C} F_{j,d}, \sigma^{\text{C}}) \tag{37}$$

where:

- μ^{K} is the log of the intercept of the rate constant parameter.
- μ^{C} is the log of the intercept of the the asymptotic concentration parameter.
- β^{K} is the slope of the rate constant parameter.
- β^{C} is the slope of the rate asymptotic concentration parameter.
- σ^{K} is the residual standard deviation of the rate constant parameter.
- σ^{C} is the residual standard deviation of the asymptotic concentration parameter.

6.2 Prior Specifications

We applied the following informative priors and truncations to the prior distributions:

$$C_{j,d} \sim \text{Normal}(6000, 2000) \tag{38}$$

$$K_{j,d} \sim \text{Normal}(0.075, 0.025), \quad \text{truncated } 0 < K_{j,d} < 0.2 \tag{39}$$

$$D_{j,d} \sim \text{Normal}(0, 1), \quad \text{truncated } 0 < D_{j,d} < 6 \tag{40}$$

$$\sigma \sim \text{Normal}(0, 400), \quad \text{truncated } \sigma > 0 \tag{41}$$

$$\sigma^{fluo} \sim \text{Inverse-Gamma}(0.4, 0.3) \tag{42}$$

$$\mu^{\text{fluo}}_{j,d} \sim \text{Uniform}(-\infty, \infty) \tag{43}$$

$$\mu^{\text{K}} \sim \text{Uniform}(-\infty, \infty) \tag{44}$$

$$\mu^{\text{C}} \sim \text{Uniform}(-\infty, \infty) \tag{45}$$

$$\beta^{K} \sim \text{Uniform}(-\infty, \infty) \tag{46}$$

$$\beta^{C} \sim \text{Uniform}(-\infty, \infty) \tag{47}$$

$$\sigma^{K} \sim \text{Inverse-Gamma}(0.4, 0.3) \tag{48}$$

$$\sigma^{C} \sim \text{Inverse-Gamma}(0.4, 0.3) \tag{49}$$

7 Figure 6B: Proportion of Active Cells

We model the proportion of active cells in the population using the same model as Figure 1C.
