# Supplementary material for: MATRIX: rapid quantification of total and active microbial cells with single-cell phenotypes for environmental microbiomes
Source: mSystems. 2026 Jul 15;11(8):e00308-26. doi: 10.1128/msystems.00308-26 (PMC13483317; doi:10.1128/msystems.00308-26)
Supplement: Supplementary file 2 [file reviewer-comments.pdf]

AMERICAN SOCIETY FOR MICROBIOLOGY | mSystems®

MATRIX: Rapid Quantification of Total and Active Microbial Cells with Single-Cell Phenotypes for Environmental Microbiomes

Milena Gonzalo, Xipeng Liu, Yann Dufour, and Ashley Shade

Corresponding Author(s): Ashley Shade, CNRS Delegation Alpes

Review Timeline:

Submission Date:	March 13, 2026
Editorial Decision:	April 20, 2026
Revision Received:	June 12, 2026
Accepted:	June 22, 2026

Editor: Ariane Peralta

Reviewer(s): Disclosure of reviewer identity is with reference to reviewer comments included in decision letter(s). The following individuals involved in review of your submission have agreed to reveal their identity: Canan Karakoc (Reviewer #1)

Transaction Report:

(Note: With the exception of the correction of typographical or spelling errors that could be a source of ambiguity, letters and reports are not edited. The original formatting of letters and referee reports may not be reflected in this compilation.)

DOI: https://doi.org/10.1128/msystems.00308-26

Re: mSystems00308-26 (**MATRIX: Rapid Quantification of Total and Active Microbial Cells with SingleCell Phenotypes for Environmental Microbiomes**)

Dear Prof. Ashley Shade:

Thank you for the privilege of reviewing your work. Below you will find my comments, instructions from the mSystems editorial office, and the reviewer comments.

The manuscript is well written and effectively addresses the challenge of quantifying microbiome responses across a range of sample types, from pure cultures to environmental bacterial isolates and soil communities. The detailed 'wet lab' workflow supports reproducibility and allows for adaptation to future studies and diverse sample types. However, the manuscript could more clearly communicate the true cost and accessibility of this workflow. As noted by another reviewer, a more balanced discussion of cost would strengthen the manuscript and highlight an important limitation, particularly for researchers working with limited resources. Additionally, there may be an opportunity for the authors to use this work to encourage adoption of these workflows by pay-for-service laboratories, which could help offset costs and support the broader application of MATRIX.

Please return the manuscript within 60 days; if you cannot complete the modification within this time period, please contact me. If you do not wish to modify the manuscript and prefer to submit it to another journal, notify me immediately so that the manuscript may be formally withdrawn from consideration by mSystems.

Revision Guidelines

To submit your modified manuscript, log into the submission site at https://msystems.msubmit.net/cgi-bin/main.plex. Go to Author Tasks and click the appropriate manuscript title to begin. The information you entered when you first submitted the paper will be displayed; update this as necessary. Note the following requirements:

• Upload point-by-point responses to the issues raised by the reviewers in a file named "Response to Reviewers," NOT in your cover letter.
• Upload a compare copy of the manuscript (without figures) as a "Marked-Up Manuscript" file.
• Upload a clean .DOC/.DOCX version of the revised manuscript and remove the previous version.
• Each figure must be uploaded as a separate, editable, high-resolution file (TIFF or EPS preferred), and any multipanel figures must be assembled into one file.
• Any supplemental material intended for posting by ASM should be uploaded with their legends separate from the main manuscript. You can combine all supplemental material into one file (preferred) or split it into a maximum of 10 files with all associated legends included.

For complete guidelines on revision requirements, see our Submission and Review Process webpage. Submission of a paper that does not conform to guidelines may delay acceptance of your manuscript.

Data availability: ASM policy requires that data be available to the public upon online posting of the article, so please verify all links to sequence records, if present, and make sure that each number retrieves the full record of the data. If a new accession number is not linked or a link is broken, provide mSystems production staff with the correct URL for the record. If the accession numbers for new data are not publicly accessible before the expected online posting of the article, publication may be delayed; please contact production staff (mSystems@asmusa.org) immediately with the expected release date.

Publication Fees: For information on publication fees and which article types are subject to charges, visit our website. If your manuscript is accepted for publication and any fees apply, you will be contacted separately about payment during the production process; please follow the instructions in that e-mail. Arrangements for payment must be made before your article is published.

ASM Membership: Corresponding authors may join or renew ASM membership to obtain discounts on publication fees. Need to upgrade your membership level? Please contact Customer Service at Service@asmusa.org.

The ASM Journals program strives for constant improvement in our submission and publication process. Please tell us how we can improve your experience by taking this quick Author Survey.

Thank you for submitting your paper to mSystems.

Sincerely,
Ariane Peralta
Editor
mSystems

Reviewer #1 (Comments for the Author):

The manuscript "MATRIX: Rapid Quantification of Total and Active Microbial Cells with Single Cell Phenotypes for Environmental Microbiomes" offers a single-cell analytics pipeline to define community size and activity. This is a well-motivated methods paper. The core problem that microbiome studies rely on relative abundance without direct cell counts or activity measurements is real and important. The workflow is practical, the Bayesian modeling is a strength, and the validation across pure cultures, isolates, and soil communities is reasonably thorough. That said, there are several places where framing and discussion of limitations could be more balanced, and some results need clearer interpretation.

General comments:

1. The authors mention fungi and archaea detection as a future direction (line 476), but it is a current limitation, saying "environmental microbiomes" in the title, while only detecting bacteria, might be an overstatement.

2. Introduction frames indirect methods (qPCR, spike-ins) almost entirely as problems to overcome, MATRIXcomplements them, instead of replacing them. DNA-based methods provide taxonomic identity, detect rare taxa below imaging thresholds, and reveal functional potential (e.g., which lineages are spore-formers, carry ARGs, form biofilms). The authors could explicitly position MATRIX as part of an integrated toolkit rather than implying it supersedes molecular approaches. Similarly, RNA-based methods (metatranscriptomics, rRNA:rDNA ratios) are established approaches for assessing active fractions that go unmentioned.

3. The introduction criticizes flow cytometry for requiring specialized instrumentation (lines 96-97), but the QUANTOM Tx counter is itself specialized, proprietary equipment. The authors should acknowledge this symmetry. The authors mention the cost per sample (~€6), which is helpful, but mentioning the capital cost of the instrument and the consumables would be a fairer comparison.

4. Statistical modeling is a strength, but underexplained. The Bayesian hierarchical approach for jointly estimating total counts and active proportions is well-motivated (lines 428-438), but the model specifications are scattered across the methods and results, making reproducibility harder. A supplementary table summarizing each model's structure, priors, and diagnostics would help.

Specific comments:

Strain choices: E. coli and P. protegens are reasonable as model organisms, but the authors should state why these two were chosen, and acknowledge that both are Gram-negative Proteobacteria. A Gram-positive organism or one with different cell envelope properties might change interpretations.

Cell eccentricity: Eccentricity is correlated with the growth faces but also could indicate cell chaining, microcolony formation, or other multicellular phenotypes. A brief discussion about how the watershed segmentation handles chains of cells, which could artifactually inflate eccentricity, would be great.

Lines 304-314: The authors show that the proportion of active P. protegens cells peak at ~70% at 6 hours and drop below 25% by 18 hours, but they don't clearly distinguish between several possible explanations: cells transitioning from active to inactive (true deactivation), active cells dying preferentially, differential staining sensitivity at different growth phases, or the accumulation of dead/inactive cells diluting the active fraction. The statement that OD and total counts are unreliable metrics for "functional size" is valid, but the mechanistic interpretation of why the active proportion declines needs more care. Is this entry into stationary-phase dormancy? Nutrient depletion reduces reductase activity below the RSG detection threshold? Since these are batch cultures in rich media, oxygen depletion is a plausible driver of declining RSG signal, specifically, given that RSG is reduced by electron transport chain reductases. The authors could discuss these possibilities.

Lines 366-367: Finding that SI = 0% across all three soils is interesting and deserves more discussion. Does this truly mean there are no vegetative dormant cells in these soils, or is this an artifact of how RSG sensitivity interacts with different dormancy states? Lysozyme resistance captures spores and thick-walled cells but misses other dormancy strategies (persister cells, VBNC states, cells in protective microaggregates). The authors could discuss the diversity of dormancy strategies and how each would be classified in their RI/RA/SA/SI framework. The claim that "most lysozyme-sensitive cells were also active" has important ecological implications. Connecting the observed fluorescence intensity distributions to known dormancy biology would be interesting.

Around Line 386: Galibier and Miribel might differ in texture, climate history, organic matter content, and baseline community composition. Without any compositional data, the ecological interpretation remains speculative. Even basic information like C:N ratio or organic matter content would help.

Lines 446-448: The ~75% {plus minus} 18% recovery is presented as acceptable, but the variance is high, and the spike-in used a single species in a single soil type. Recovery will likely vary systematically with cell morphology (filamentous vs. coccoid), cell

wall properties (Gram-positive vs. Gram-negative), attachment strength (biofilm-formers vs. planktonic cells), and soil physicochemistry. Authors could acknowledge some of these limitations.

Lines 462-463: The discussion acknowledges that reductases persist after cell death, but the window of detectability ("a few hours") is vague. For the soil experiments, the time between extraction and measurement matters and should be reported explicitly and consistently.

Figure 1: Panel A uses dual y-axes with three overlaid data series, but the scaling relationship between the axes is arbitrary and visually implies a correspondence that Panel B then shows is nonlinear and species-specific. The figure would be clearer if Panel A showed OD and cell counts as separate sub-panels, or if the dual axes were more carefully justified. The figure legend says "optical density and counts are scaled on two different axes" but doesn't explain the scaling choice.

Figure 3: Do the isolates with wide CrIs have lower total counts? If so, the uncertainty is expected, but it should be noted that the method's precision for estimating active proportions degrades at lower cell densities.

Figure 4: Was the model also run without the non-negativity constraint to see what the unconstrained SI estimate would be? A small negative value within the uncertainty of zero would be reassuring, while a strongly negative value might indicate model misspecification.

Figure 5: With only 3 sites × 2 seasons × 3 time points = 18 observations, the linear fit is driven by relatively few points. How robust is this relationship to removing any single site or season? For example, if the Miribel 7 warm-season points (which appear to be the highest values driving the upper end) were excluded, would the relationship hold?

Figure 6: Interpretation could benefit from showing the data as fold-change or effect size rather than raw values, since the two soils start at different baselines. Also, panel C shows that RSG fluorescence distributions are essentially identical across all conditions, which the authors interpret as meaning both soils support similar activity levels. But an alternative interpretation is that RSG intensity per cell is relatively insensitive to the heat treatment, and the real signal is in the proportion of active cells (panel B). This distinction matters for what RSG is actually measuring.

Reviewer #2 (Comments for the Author):

This is a Methods paper describing a pipeline for enumerating cell contents in microbial communities. Two different dyes discriminante between total number of cells and the proportion of active cells. The authors argue that this pipeline (MATRIX) oír similar alternative pipelines would lead to a better resolution in a variety of microbial ecology experiments and promote a better understanding of the ecological function. In my opinión, this approavch may be of interest to microbial ecologists addressing a variety of questions. The paper is well written, and the pipeline is tested on pure cultures as well as on soil simple communities. I only have a few points to make.

Major concern

The heat stress experiment produced results that sort of contradict the general understanding of heat stress response. Typically cells shut down to protect DNA and protein to at least a degree. Your results show increase in cell content, sort of stimulation rather than shut down. What is your explanation for this? I found not a word of discussion.

Minor

L 150. Incomplete sentence, please rewrite

L 198. Should it be either SYBR or RSG?

L 709. Should be categories

Fig 1A. OD not shown for H 18 for E. Coli

Fig 3A. Paenibacillus has too many replicates when only 3 are mentioned for each strain

>>>We thank the editor and two reviewers for their constructive comments. We have addressed each numbered comment in the tracked-changes revision; each change is labeled with the corresponding comment number. To find where a comment was addressed, use Find (Ctrl+F) to search the comment number. Line and page references in our responses correspond to the tracked-changes document.

Editor comments

E1
The manuscript is well written and effectively addresses the challenge of quantifying microbiome responses across a range of sample types, from pure cultures to environmental bacterial isolates and soil communities. The detailed 'wet lab' workflow supports reproducibility and allows for adaptation to future studies and diverse sample types. However, the manuscript could more clearly communicate the true cost and accessibility of this workflow. As noted by another reviewer, a more balanced discussion of cost would strengthen the manuscript and highlight an important limitation, particularly for researchers working with limited resources. Additionally, there may be an opportunity for the authors to use this work to encourage adoption of these workflows by pay-for-service laboratories, which could help offset costs and support the broader application of MATRIX.

>>>Thank you for editing the manuscript and for the positive comments. We have made changes to communicate the cost of the workflow by adding the cost of the instrument and improved clarity in explanation of the consumables. We like the idea about pay-for-service laboratories to help to offset costs and support the broader application and have also added this to the discussion section. We have also clarified that the image analyses components of the workflow are transferrable to any microscope images and that the QUANTOM instrument is not required.

Reviewer #1 (Comments for the Author):

R1.0
The manuscript "MATRIX: Rapid Quantification of Total and Active Microbial Cells with Single Cell Phenotypes for Environmental Microbiomes" offers a single-cell analytics pipeline to define community size and activity. This is a well-motivated methods paper. The core problem that microbiome studies rely on relative abundance without direct cell counts or activity measurements is real and important. The workflow is practical, the Bayesian modeling is a strength, and the validation across pure cultures, isolates, and soil communities is reasonably thorough. That said, there are several places where framing and discussion of limitations could be more balanced, and some results need clearer interpretation.

>>>Thank you for your time in reviewing the manuscript and for your thoughtful comments. We appreciate the comment about the limitations and have addressed them by expanding the discussion section to include more precise costs and details about the consumables.

General comments:

R1.1
1. The authors mention fungi and archaea detection as a future direction (line 476), but it is a current limitation, saying "environmental microbiomes" in the title, while only detecting bacteria, might be an overstatement.

>>>Thank you, we prefer to retain the title to keep the more general MATRIX acronym/name because we have ongoing work with the same workflow targeting the other lineages and this is just the first report of the method. However, we have updated the abstract to specify bacterial counts are treated in this work.

R1.2
2. Introduction frames indirect methods (qPCR, spike-ins) almost entirely as problems to overcome, MATRIX complements them, instead of replacing them. DNA-based methods provide taxonomic identity, detect rare taxa below imaging thresholds, and reveal functional potential (e.g., which lineages are spore-formers, carry ARGs, form biofilms).The authors could explicitly position MATRIX as part of an integrated toolkit rather than implying it supersedes molecular approaches. Similarly, RNA-based methods (metatranscriptomics, rRNA:rDNA ratios) are established approaches for assessing active fractions that go unmentioned.

>>>Thank you for this comment, which we appreciate because this is how we intend to use MATRIX- as a complement to DNA-based sequencing, and we didn't mean to imply that it should replace molecular approaches. We have made changes to the introduction to clarify this complementation.

>>>The reviewer is correct in that we do not mention the rRNA:rDNA methods because our focus is on quantifying the number of active individual cells (community or population size) and this method does not provide this information. We do mention metatranscriptomics.

R1.3
3. The introduction criticizes flow cytometry for requiring specialized instrumentation (lines 96-97), but the QUANTOM Tx counter is itself specialized, proprietary equipment. The authors should acknowledge this symmetry. The authors mention the cost per sample (~€6), which is helpful, but mentioning the capital cost of the instrument and the consumables would be a fairer comparison.

>>>Thank you for this comment. We now clarify that the MATRIX image analysis and modeling can be applied to any type of microscope image, and is not reliant on the QUANTOM Tx. To automate image acquisition, the QUANTOM Tx is a benchtop equipment similar in price and footprint to "small equipment" that is maintained by a single lab/PI without specialized expertise, core facility requirements or maintenance contract. We have augmented information about the flexibility of the pipeline and cost of the QUANTOM and the precise consumables in the Discussion.

R1.4
4. Statistical modeling is a strength but underexplained. The Bayesian hierarchical approach for jointly estimating total counts and active proportions is well-motivated (lines 428-438), but the model specifications are scattered across the methods and results, making reproducibility harder. A supplementary table summarizing each model's structure, priors, and diagnostics would help.

>>>Thank you, we have added Supplementary Information 1 and describe in detail there the models and prior specifications.

Specific comments:
R1.5
Strain choices: E. coli and P. protegens are reasonable as model organisms, but the authors should state why these two were chosen, and acknowledge that both are Gram-negative

Proteobacteria. A Gram-positive organism or one with different cell envelope properties might change interpretations.

>>>Thank you for this comment, good point. We have added a sentence motivating our use of these organisms.

>>>The manufacturer has applied the QUANTOM protocol to a variety of bacteria and they offer strain-specific staining profiles (including G+ like *Lactobacillus*, *Bacillus*) in their "Cell Database" (https://logosbio.com/image-gallery/?image_gallery_category=quantom-tx) with associated technical information on their website. We are confident from the manufacturer's information and from our own work with environmental strains that the Quantom assay is robust and that population- and community-level interpretations are unlikely to be affected.

R1.6
Cell eccentricity: Eccentricity is correlated with the growth faces but also could indicate cell chaining, microcolony formation, or other multicellular phenotypes. A brief discussion about how the watershed segmentation handles chains of cells, which could artifactually inflate eccentricity, would be great.

>>>Thank you, we have added supplementary images (Fig S1) to demonstrate the accuracy of the segmentation when cells are clustered or forming chains.

R1.7
R1.7.1
Lines 304-314: The authors show that the proportion of active P. protegens cells peak at ~70% at 6 hours and drop below 25% by 18 hours, but they don't clearly distinguish between several possible explanations: cells transitioning from active to inactive (true deactivation), active cells dying preferentially, differential staining sensitivity at different growth phases, or the accumulation of dead/inactive cells diluting the active fraction.

>>>Thank you for this detailed comment. The proportional activity data is directly related to the total and active count data shown the above panel 1B. Therefore, the proportion of inactive cells is most likely a combination of cell dying or becoming inactive as the population density increases.

- In the case of inactive but not dead cells, the total counts should be maintained while the active counts should decrease. Figure 1B shows that the total counts slightly increased while the active counts slightly decreased. This suggests that there was some deactivation among the previously active component because there was not a large drop in the number of cells.
- In the case of differential staining sensitivity at different growth phases, we did not test this directly, but the literature shows that the stain is suitable across growth stages in lab cultures. We have added a specific reference in the discussion (Baert et al. 2016) to convey this information.
- In the case of the accumulation of dead cells that "dilutes" the active fraction, dead cells would not be included in the total (SYBR) counts unless their membranes were uncompromised. Therefore, the number of dead cells remains unobserved because we cannot measure it directly. However, as for point 1, because the total counts increases slightly while active counts decreases, we can deduce that substantial overall loss of cells is not a major driver of the observed decrease in activity.

R1.7.2
The statement that OD and total counts are unreliable metrics for "functional size" is valid, but the mechanistic interpretation of why the active proportion declines needs more care. Is this entry into stationary-phase dormancy? Nutrient depletion reduces reductase activity below the RSG detection threshold? Since these are batch cultures in rich media, oxygen

depletion is a plausible driver of declining RSG signal, specifically, given that RSG is reduced by electron transport chain reductases. The authors could discuss these possibilities.

>>>Thank you for this comment. We cannot know directly the mechanisms as to why the RSG signal decreased in our experiment because we did not measure it. However, this has been explored in the literature and we have added a reference (Baert et al. 2013) that shows RSG signal responsiveness to changes in growth conditions like oxygen and nutrient depletion that would decrease metabolism.

R1.8
Lines 366-367: Finding that SI = 0% across all three soils is interesting and deserves more discussion. Does this truly mean there are no vegetative dormant cells in these soils, or is this an artifact of how RSG sensitivity interacts with different dormancy states? Lysozyme resistance captures spores and thick-walled cells but misses other dormancy strategies (persister cells, VBNC states, cells in protective microaggregates). The authors could discuss the diversity of dormancy strategies and how each would be classified in their RI/RA/SA/SI framework. The claim that "most lysozyme-sensitive cells were also active" has important ecological implications. Connecting the observed fluorescence intensity distributions to known dormancy biology would be interesting.

>>>Thank you for your comment. We revisited the Figure 4 model and analysis and relaxed the constraints to better estimate the four categories. Although the SI category remains small, it is not necessarily zero; our revised estimate places it between 0% and 20%. As a result, the posterior estimates for each category are somewhat less precise. We have expanded the discussion accordingly and added a detailed description of the model in supplementary materials.

>>>We agree with the reviewer and appreciate the ecological implications of the different dormancy strategies and the framework available given the data provided by the MATRIX pipeline. It is a very important question that we believe needs to be addressed with much larger sample size than what we present here, which is simply to convey the potential of the method. We are pursuing this research now with a variety of soils, but it is not complete enough to include in this methods paper and would also likely distract from the communication of the method and get more into the scientific questions. Therefore, we ask the reviewer to stay tuned as we explore this phenomenon more fully in subsequent work.

R1.9
Around Line 386: Galibier and Miribel might differ in texture, climate history, organic matter content, and baseline community composition. Without any compositional data, the ecological interpretation remains speculative. Even basic information like C:N ratio or organic matter content would help.

>>>Thank you for this comment, and we agree that these soils vary in many physical-chemical parameters, including C:N ratio. We had previously reported some of these values (like texture, pH, moisture) in Table 2, and have now also added C:N ratio and organic matter content.

>>>We have also tempered the sentence to convey that uncertainty in the mechanism of the response.

R1.10
Lines 446-448: The ~75% {plus minus} 18% recovery is presented as acceptable, but the variance is high, and the spike-in used a single species in a single soil type. Recovery will likely vary systematically with cell morphology (filamentous vs. coccoid), cell wall properties

(Gram-positive vs. Gram-negative), attachment strength (biofilm-formers vs. planktonic cells), and soil physicochemistry. Authors could acknowledge some of these limitations.

>>>Thank you for this comment. We agree. We note that we had previously discussed the limitation of cell morphology (filamentous vs. coccoid): *"Cell extraction is also influenced by cell morphology and density, potentially biasing against organisms with low-density cell structures or mycelial forms (36)."* We had also previously discussed the limitation of potential differences across different soils: *"Reported extraction efficiencies vary across soils (33)(18)"* and have now further added to clarify *"likely due to differences in physico-chemical properties."* However, we did not originally mention the remaining limitations relating to cell wall properties and attachment strength. We have now added three new references and expanded discussion of these limitations as well as protocol recommendations for optimizing recovery of attached cells. Finally, we have also added a new S. Fig 2 showing more details of our recovery of cells from subsequent soil blendings, as described already in the methods. This protocol's recovery of cells is in line with or exceeds the what is typically recovered from soils in the literature.

R1.11
Lines 462-463: The discussion acknowledges that reductases persist after cell death, but the window of detectability ("a few hours") is vague. For the soil experiments, the time between extraction and measurement matters and should be reported explicitly and consistently.

>>>Thank you, we agree and have expanded the discussion around this topic, adding the need to report processing times, adding references from the literature regarding RSG decay, and including a new note from our own experiences with RSG decay in *E.coli* cultures. We note that there are many different ways for environmental cells to die *in situ*, and that the RSG signal decay will be very likely a function of the mechanism of death.

R1.12
Figure 1: Panel A uses dual y-axes with three overlaid data series, but the scaling relationship between the axes is arbitrary and visually implies a correspondence that Panel B then shows is nonlinear and species-specific. The figure would be clearer if Panel A showed OD and cell counts as separate sub-panels, or if the dual axes were more carefully justified. The figure legend says "optical density and counts are scaled on two different axes" but doesn't explain the scaling choice.

>>>Thank you for this comment. We clarify that by "scaled" we simply mean "presented," and that no data modification has been performed to make the counts and OD series look any more or less compatible with each other. Furthermore, we directly show (and discuss in the text) their nonlinear relationship in Figure 1B. The axes ranges were not arbitrary but selected based on the minimum and maximum observed values in these series so that they could by fully captured. We have added this information to the Figure 1 legend.

>>>We chose to use dual axes to enable comparisons of the two strains' different values with standardized y-axis ranges, which allows readers to view directly how similar ODs have different cell concentrations across strains.

R1.13
Figure 3: Do the isolates with wide CrIs have lower total counts? If so, the uncertainty is expected, but it should be noted that the method's precision for estimating active proportions degrades at lower cell densities.

>>>Thanks for the question. We specifically investigated this and found no evidence that larger CrIs are related to lower cell counts, or that precision degrades at lower densities. The CrI reflects variability between biological replicates more than uncertainty in cell counts.

>>>Considering Fig 3, we note that the CrIs are generally small. Cell density is very high in these data because they are showing counts from pure cultures.

R1.14
Figure 4: Was the model also run without the non-negativity constraint to see what the unconstrained SI estimate would be? A small negative value within the uncertainty of zero would be reassuring, while a strongly negative value might indicate model misspecification.

>>>We revised the model to better reflect the experimental design and physical constraints on the system. With the new model, none of the four categories are strictly zero. The proportion of the SI category remains small and consistent across the sampling locations. The non-negative constraint is part of the model to ensure that proportions stay between zero and one as required by the laws of probabilities. The figure and text have been revised accordingly.

R1.15
Figure 5: With only 3 sites × 2 seasons × 3 time points = 18 observations, the linear fit is driven by relatively few points. How robust is this relationship to removing any single site or season? For example, if the Miribel 7 warm-season points (which appear to be the highest values driving the upper end) were excluded, would the relationship hold?

>>>Thanks for this question. The Bayesian model updating provides the full posterior probability distribution of the estimated parameters, and therefore the robustness of the conclusion, given the model and the data. The priors we chose for this model are noninformative. Generally, in the Bayesian framework there is not rational for performing post-hoc robustness analyses like bootstrapping or jack-knifing used in the classical framework because of this updating of the full posterior probability distribution.

R1.16.1
Figure 6: Interpretation could benefit from showing the data as fold-change or effect size rather than raw values, since the two soils start at different baselines.

>>>Thanks for this suggestion. The fold change given the heat treatment is provided in the text. We have now made this more complete by adding their CrIs. The relative change of proportion of active cells is also shown in panel B (to accompany panel A).

R1.16.2
Also, panel C shows that RSG fluorescence distributions are essentially identical across all conditions, which the authors interpret as meaning both soils support similar activity levels. But an alternative interpretation is that RSG intensity per cell is relatively insensitive to the heat treatment, and the real signal is in the proportion of active cells (panel B). This distinction matters for what RSG is actually measuring.

>>>Thanks for this comment. We agree that the proportion of active cells is the responsive signal in this experiment, as shown in Fig 6B, and realized that the wording was not clear in the text. We have now clarified “activity levels *per cell.*”

Reviewer #2 (Comments for the Author):

R2.0
This is a Methods paper describing a pipeline for enumerating cell contents in microbial communities. Two different dyes discriminante between total number of cells and the proportion of active cells. The authors argue that this pipeline (MATRIX) oír similar alternative pipelines would lead to a better resolution in a variety of microbial ecology experiments and promote a better understanding of the ecological function. In my opinion, this approach may be of interest to microbial ecologists addressing a variety of questions. The paper is well written, and the pipeline is tested on pure cultures as well as on soil simple communities. I only have a few points to make.

>>>Thank you for your time in reviewing the piece and for your thoughtful comments. We're glad you agree that MATRIX will be useful.

Major concern

R2.1
The heat stress experiment produced results that sort of contradict the general understanding of heat stress response. Typically cells shut down to protect DNA and protein to at least a degree. Your results show increase in cell content, sort of stimulation rather than shut down. What is your explanation for this? I found not a word of discussion.

>>>Thank you for this question. We agree that the term "heat stress" was too broad for the community-level response we observed. In diverse soil communities, warming is unlikely to affect all taxa similarly: some populations may reduce activity, while others with higher thermal tolerance may maintain or increase it. We therefore interpret the increase in active cell content as a shift in community response, likely reflecting selection for heat-tolerant taxa and higher respiration among those populations at the treatment temperature, rather than a uniform stimulatory effect on all cells. We have revised the results section to clarify this interpretation, use more precise language about "stress," and cite work showing that warming often causes an initial increase in soil respiration followed by attenuation or decline depending on context.

New references cited:
https://www.sciencedirect.com/science/article/abs/pii/S0038071716308331
https://www.sciencedirect.com/science/article/abs/pii/S1369527424001346

Minor
R2.2
L 150. Incomplete sentence, please rewrite
>>> Apologies for the oversight, done.

R2.3
L 198. Should it be either SYBR or RSG?
>>Yes. Corrected.

R2.4
L 709. Should be categories
>>>We think that this comment is referring to the legend of Figure 5 L109 which reads *"fluorescence. B) Initial substrate concentration as a function of total RSG fluorescence. The"*. It is unclear to us what should be categories?

R2.5
Fig 1A. OD not shown for H 18 for E. Coli

>>>Thank you, this is accurate. We have missing OD data for 16h for both cultures. This was due to a user error at this collection time point for the OD. We have added this note to the methods.

R2.6
Fig 3A. Paenibacillus has too many replicates when only 3 are mentioned for each strain
>>> Thank you for catching this, we appreciate it! There were two distinct *Paenibacillus* strains that had the same taxonomy label, but they were accidentally aggregated into one in Fig. 3 because of overlap in their labeling. We have corrected this to separate the two strains and updated Figure 3. No conclusions are changed from this update.

Re: mSystems00308-26R1 (**MATRIX: Rapid Quantification of Total and Active Microbial Cells with Single-Cell Phenotypes for Environmental Microbiomes**)

Dear Prof. Ashley Shade:

Thank you for your careful and thorough attention to the reviewer feedback. The authors have done an excellent job addressing the comments in a detailed manner.

Your manuscript has been accepted, and I am forwarding it to the ASM production staff for publication. Your paper will first be checked to make sure all elements meet the technical requirements. ASM staff will contact you if anything needs to be revised before copyediting and production can begin. Otherwise, you will be notified when your proofs are ready to be viewed.

Data Availability: ASM policy requires that data be available to the public upon online posting of the article, so please verify all links to sequence records, if present, and make sure that each number retrieves the full record of the data. If a new accession number is not linked or a link is broken, provide production staff with the correct URL for the record. If the accession numbers for new data are not publicly accessible before the expected online posting of the article, publication may be delayed; please contact ASM production staff immediately with the expected release date.

Publication Fees: For information on publication fees and which article types have charges, please visit our website. We have partnered with Copyright Clearance Center (CCC) to collect author charges. If fees apply to your paper, you will receive a message from no-reply@copyright.com with further instructions. For questions related to paying charges through RightsLink, please contact CCC at ASM_Support@copyright.com or toll free at +1-877-622-5543. CCC makes every attempt to respond to all emails within 24 hours.

ASM Membership: Corresponding authors may join or renew ASM membership to obtain discounts on publication fees. Need to upgrade your membership level? Please contact Customer Service at Service@asmusa.org.

Embargo Policy: A press release may be issued as soon as the manuscript is posted on the mSystems Latest Articles webpage. The corresponding author will receive an email with the subject line "ASM Journals Author Services Notification" when the article is available online.

Cover Image Submissions: If you would like to submit a potential Cover Image, please email a file and a short legend to msystems@asmusa.org. Please note that we can only consider images that (i) the authors created or own and (ii) have not been previously published. By submitting, you agree that the image can be used under the same terms as the published article. Image File requirements: TIF/EPS, 7.5 inches wide by 8.25 inches tall (at least 2,250 pixels wide by 2,475 pixels tall), minimum 300 dpi resolution (600 dpi preferred), RGB, and no figure elements, e.g., arrows or panel labels. The legend should be a short description of the image, 1-2 sentences recommended. Please download and use this interactive template in Adobe to ensure that your proposed cover image meets our size requirements (https://journals.asm.org/pb-assets/pdf-text-excel-files/ASM-Interactive-Sizing-Cover-Template-1715689791.pdf).

Thank you for submitting your paper to mSystems.

Sincerely,
Ariane Peralta
Editor
mSystems
